# An Unusual Source of Cerebral Embolism Caused by Lambl’s Excrescences

**DOI:** 10.7759/cureus.40910

**Published:** 2023-06-24

**Authors:** Narek Hakobyan, Nosakhare Ilerhunmwuwa, Mustafa Wasifuddin, Fares Jamal, Tatyana Zagoruychenko

**Affiliations:** 1 Internal Medicine, Brookdale University Hospital Medical Center, New York City, USA

**Keywords:** aortic valve, stroke, covid-19, embolic stroke, lambl's excrescence

## Abstract

Lambl's excrescences (LEs) are delicate filiform strands formed by connective tissue located along the valve closure lines within the cardiovascular system. Most cases of these excrescences manifest without discernible symptoms, and the exact etiological factors contributing to their formation remain unknown. These excrescences may embolize to the brain, causing strokes. It is essential that all other possible causes of stroke be eliminated prior to identifying Lambl's excrescences as the cause of the stroke. Herein, we present a case of a patient who suffered a stroke, and all conventional testing for common causes of embolic strokes was ruled out. In pursuit of a comprehensive evaluation encompassing the classification of the stroke as cryptogenic, a transesophageal echocardiogram (TEE) was performed, which effectively disclosed the existence of LEs situated on the aortic valve leaflets. The patient was treated with anticoagulation and discharged with close follow-up monitoring. To culminate, the inclusion of this case within our study augments the currently scarce pool of instances that exhibit such characteristic cardioembolic phenomena, thereby accentuating the necessity for additional prospective investigations to substantiate the existence of a causative association linking LEs and cardioembolic strokes.

## Introduction

Stroke is the leading cause of long-term disability and the second leading cause of death worldwide, resulting in the death of 26 million people each year [1]. A stroke can be caused by hemorrhage in one-third of cases and ischemia in two-thirds of cases. The latter may be caused by blockages in small blood vessels of the brain, arterial embolisms from cardiac sources, or atherosclerosis of the cerebral circulation [1]. Heart-related causes account for 14%-30% of ischemic strokes [2]. Over the past few decades, stroke incidence has decreased overall; however, strokes caused by cardiac sources have doubled [1]. Stroke incidence is significantly influenced by improper management of modifiable risk factors such as hypertension, diabetes, smoking, arrhythmias, or hypercholesterolemia [3]. The severity of brain damage caused by stroke may depend on the length and severity of the ischemia as well as the presence of collaterals [4].

Lambl's excrescences were first described as a cause of embolic stroke in 1856 by Vilém Dušan Lambl, a Bohemian physician [2]. In the hearts of most people, Lambl's excrescences consist of filiform structures with rolling motion located mostly on the atrial or ventricular sides of the mitral or aortic valves. The cause is unknown but mostly attributed to wear and tear of the valve due to aging [2]. Transesophageal echocardiography is the only reliable method of detecting filiform structures [5]. Strokes caused by Lambl's excrescences are generally treated with anticoagulation, but if a second stroke occurs from the same source, an operation should be performed to remove the excrescences [6].

## Case presentation

A 53-year-old female presented to the emergency department (ED) with weakness in her left upper and lower extremities, a drooping left side of her face, and difficulty speaking. She did not have a significant medical history or a surgical history, nor had she traveled recently. According to the patient, she has never smoked, consumed alcohol, or used illicit drugs. The physical examination was unremarkable except for the neurological examination, which showed dysarthria, facial asymmetry, and weakness in the left lower and upper extremities. The National Institutes of Health Stroke Scale (NIHSS) score was 11 (with 4+ left motor arm, 4+ left motor leg, 1+ limb ataxia, 1+ sensory, and 1+ for extinction and attention). The patient was out of the tissue plasminogen activator (tPA) window.

**Figure 1:**
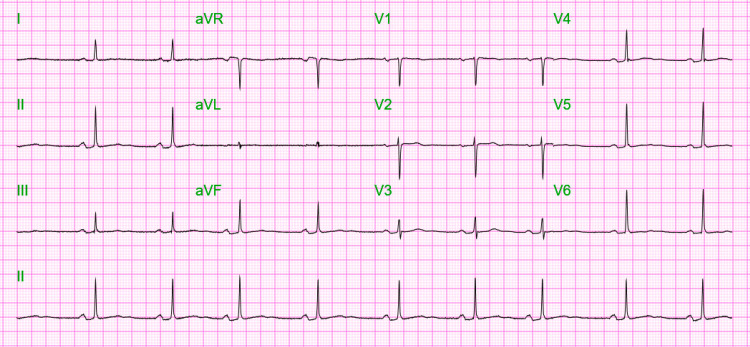
Initial electrocardiography showing sinus bradycardia

The patient's laboratory results were not significant at the time of admission. The urine toxicology screen was negative, and the lipid panel was within the reference range. Electrocardiography (EKG) showed sinus bradycardia with a normal rhythm (Figure 1).

**Figure 2:**
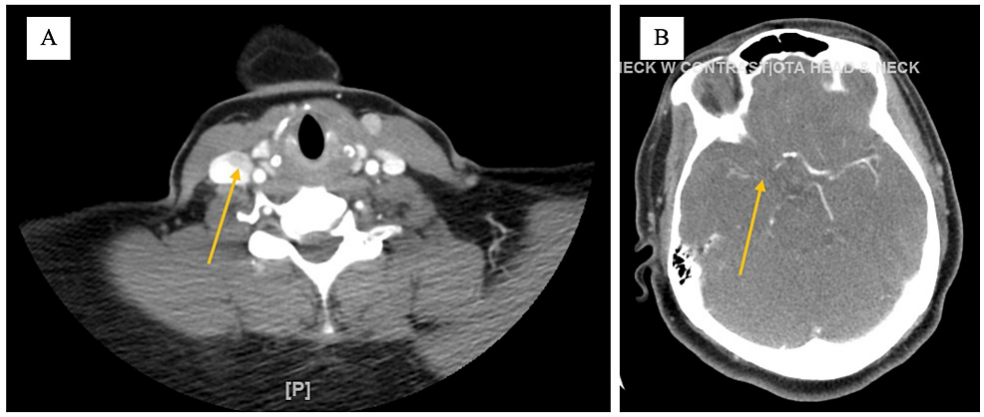
CT angiogram CT angiogram showing (A) occlusion of the supraclinoid internal carotid artery on the right (yellow arrow) and (B) cross-filling of the right A2 segment and the distal M1 segment of the middle cerebral artery (MCA) (yellow arrow).

CT scan of the head without contrast was performed, which indicated no acute intracranial pathology. A CT angiogram with contrast of the head and neck revealed occlusion of the supraclinoid internal carotid artery on the right, as well as cross-filling of the right A2 segment and the distal M1 segment of the middle cerebral artery (MCA) as shown in Figure 2. 

The patient was immediately taken for percutaneous transluminal mechanical thrombectomy of the right MCA, followed by treatment with aspirin, clopidogrel, and deep vein thrombosis (DVT) prophylaxis with heparin. After the procedure, a CT scan of the head without contrast revealed an acute infarct in the right frontal lobe and right basal ganglia. Additionally, a mass effect was observed on the right lateral ventricle without a shift in the midline. Following the procedure, the patient's left lower extremity weakness and left upper extremity weakness significantly improved; however, facial droop remains.

**Figure 3:**
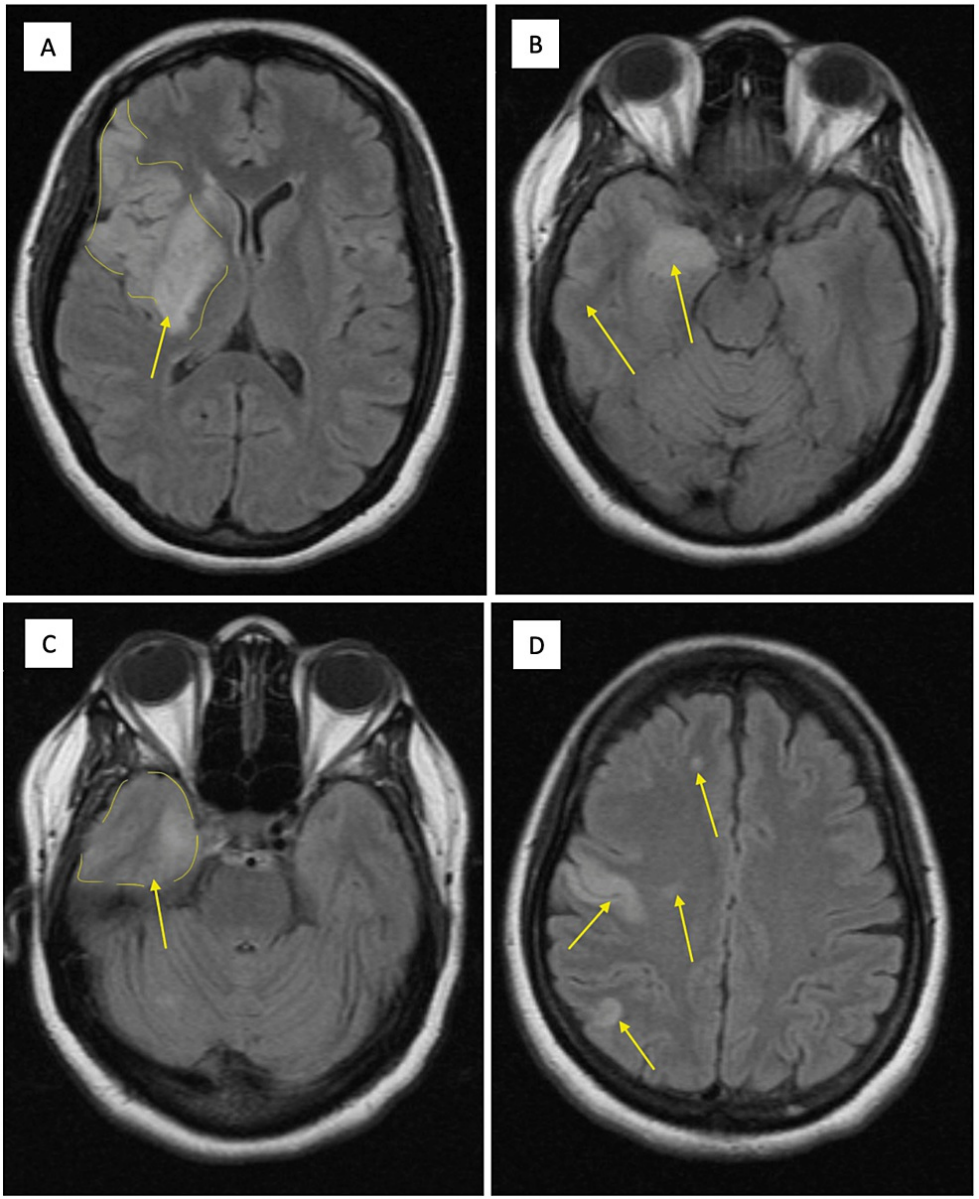
Magnetic resonance imaging Magnetic resonance imaging showing an ischemic stroke on the right side of the brain is shown in yellow arrows (A, B, C, D).

A radiological and hematological examination was conducted to identify the source of the embolus. A transthoracic echocardiogram (TTE) demonstrated a normal left ventricle size and left ventricle thickness above the normal range. During early diastole, the left ventricle displayed decreased relaxation. The TTE revealed no evidence of clots in the heart. A brain MRI without contrast was performed, which showed acute stroke in multifocal areas within the right cerebral hemisphere primarily in the inferior frontal lobe (Figure 3).

**Figure 4:**
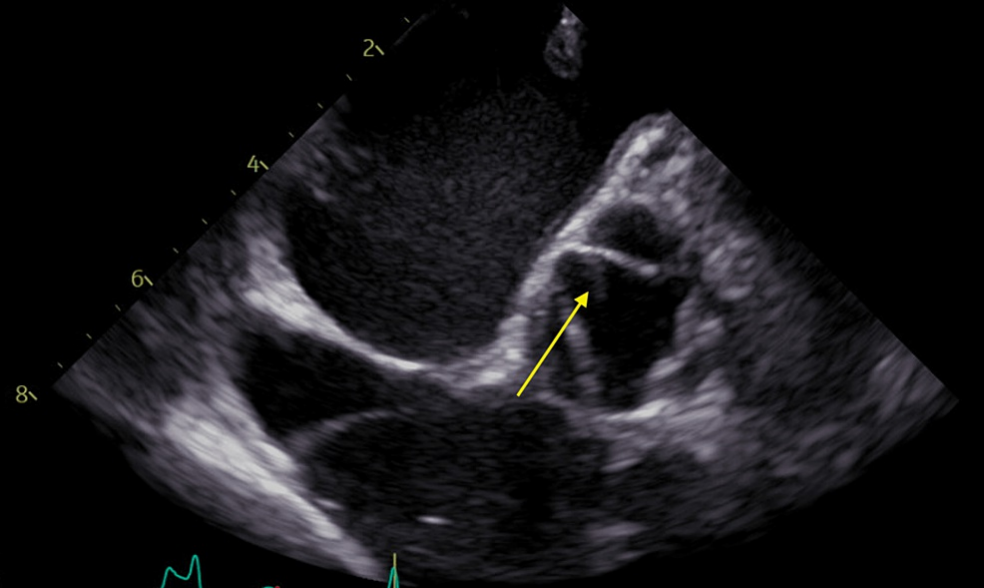
Transesophageal echocardiogram showing fine filamentous strand (yellow arrow) on the aortic valve

A bubble study was performed and revealed a small patent foramen ovale. There were negative results for bilateral Doppler ultrasounds of the lower extremities, bilateral renal ultrasounds, and a Holter study of the lower extremities. All coagulation tests, including factor V Leiden, lupus anticoagulant, anticardiolipin, antithrombin III activity, protein C and S resistance, prothrombin G20210A mutation, anti-nuclear antibody (ANA), and homocysteine levels, were negative. A transesophageal echocardiogram (TEE) was performed which revealed a fine filamentous strand (6 mm in length and 1 mm in thickness) on the aortic valve consistent with Lambl's excrescence (Figure 4), a rare cause of stroke.

The patient was hospitalized for 14 days, and the remainder of his stay was uneventful. The patient was discharged on a regimen of aspirin, ticagrelor, and atorvastatin. The patient was discharged to a rehabilitation facility for acute rehabilitation.

## Discussion

The first description of Lambl's excrescences (LEs) came from Bohemian physician Vilém Dušan Lambl. Initially, these lesions were identified on the aortic valve as filiform lesions [7]. While LE by itself is not considered to be a rare finding, LE embolizing to different sites is considered a rare condition [8]. Whenever a patient presents with an ischemic stroke, LE should be considered as a differential diagnosis [7]. In addition to ischemic stroke, LE has been linked to acute coronary syndrome as well as migrainous headaches [9]. As of 2020, 22 cases of embolized LE have been reported [8]. A stroke caused by LE occurs in one out of 200 people per year [10]. Adults are more likely to be diagnosed with LE than children, men are more likely to be diagnosed with LE than women, and the prevalence of LE increases with age, peaking between the ages of 61 and 70. A reduction in LE detection occurs after the age of 70 as a consequence of the calcification caused by aging, which makes LE detection more challenging [11]. Patients undergoing TEE are found to have valvular strands in 5.5% of cases, but the percentage increases to 40% in patients presenting with cryptogenic strokes [11].

The aortic valve and mitral valve represent the two most common locations for LE, with the aortic valve being the chief source of stroke in LE [7]. In a study conducted by Magarey et al., it was shown that LE is found in 85% of mitral valves and 2% of aortic valves [12]. The presence of LE on valves without any pathological process has also been demonstrated [13]. Nevertheless, Freedberg et al. and Roldan et al. found that LE is more common in cases of the mitral valve than in cases of the aortic valve [5,14]. In most cases, LEs are caused by wear and tear to the valve caused by aging [7]. The continuous flow of blood across the valves causes tears in the endothelium [9]. A fibrin layer is formed over the tears as a result of this process [9]. The high pressure on the left side of the heart will cause thrombi to become fibrinous flat scars. As a last step in the process, a single endothelial layer is formed on top of the fibrous surface [9]. On histological examination, LEs are composed of hyalinized and fibroelastic structures covered by an endothelial lining [7]. The most common method of detecting Lambl's excrescences is TEE, which has a sensitivity of 68% and a specificity of 85% [7,9].

It is possible for LE to appear in a variety of shapes (single strands, clusters, or rows) as well as a variety of sizes (1-10 mm in length and 1 mm in thickness) [9]. It is possible to confuse Lambl's excrescence with fibroelastoma (FE), but there are several ways in which the two can be distinguished. It is common for FE to be larger than LE, and FE is often characterized by finger-like projections that are absent in LE [9]. FEs are found on the midportion of the valve leaflet, whereas LEs are found on the valve closure sites [9]. Lastly, FE would have multiple layers of endothelium, whereas LE has a single layer [9].

A study conducted by Roldan et al. and Cohen et al. found that LE may not be a cause of cardioembolic strokes, may be present in normal valves without any pathological process, and may not require treatment [5,13]. This could be a normal variant because the blood flows continuously across the valves, and this could be supported by the fact that LEs are mostly observed on the left side of the heart due to increased pressure. According to Cohen et al. [13], stroke rates were similar in LE patients and normal valve patients in a case-control study. As well, Homma et al. [15] concluded that LEs were not a predictor of recurrent strokes. On the other hand, in a study done by Freedberg et al., they found that patients with LE have a high prevalence of cardioembolic stroke compared to patients without LE [14]. As of now, there is no clear evidence for an association between the size of LE and the risk of stroke [7].

To date, no definitive treatment has been identified for LE. A patient's presentation will determine the type of treatment to be administered [9]. Antiplatelet therapy may be recommended by some physicians in the case of asymptomatic patients, while others recommend observation only [9]. In the event of a first stroke, and after all other possible causes have been eliminated, physicians recommend the use of antiplatelet or anticoagulation therapy [9] after the stroke. Surgical intervention may be recommended in the event of a recurrent stroke when no other cause has been identified [9]. Before undergoing any surgical procedure, the patient should be started on anticoagulation along with warfarin if he/she was on antiplatelet therapy and had recurrent strokes [2]. As of now, surgery has not shown a guaranteed benefit for LE management [7]. According to Cohen et al. [13], treatment with antiplatelets, anticoagulation, or surgery had no effect on recurrent stroke risk. 

Lambl's excrescence continues to be a very rare cause of cardioembolic stroke without an established treatment. Herein, we present a patient with an established stroke confirmed to have Lambl’s excrescence. A combination of medical and surgical management may be beneficial; however, more research is needed to determine the effectiveness of this approach.

## Conclusions

We present an exceptional case report of Lambl's excrescences (LEs) in a patient who had suffered an ischemic stroke. This singular case serves as an illustrative example, underscoring the requirement for further prospective investigations aimed at establishing a definitive association between LE and cardioembolic strokes. The importance of such research endeavors cannot be overstated, since LE has the potential to cause recurrent ischemic events leading to lasting disability and irreversible damage. The significance of elucidating this relationship lies in its potential to enhance our understanding of the pathophysiological mechanisms at play. This will also enable clinicians to develop targeted interventions and ultimately improve patient outcomes.
